# Public health policy and walking in England—analysis of the 2008 ‘policy window’

**DOI:** 10.1186/s12889-015-1915-y

**Published:** 2015-07-05

**Authors:** Karen Milton, Jonathan Grix

**Affiliations:** British Heart Foundation Centre on Population Approaches for Non-Communicable Disease Prevention, Nuffield Department of Population Health, University of Oxford, Old Road Campus, Oxford, OX3 7LF UK; School of Sport, Exercise and Rehabilitation Sciences, University of Birmingham, Edgbaston, Birmingham, B15 2TT UK

**Keywords:** Public health, Policy, Walking, England, Multiple Streams

## Abstract

**Background:**

Although the government in England has a long-standing interest in walking promotion, this has not been accompanied by a coherent strategic plan or investment to support physical activity behaviour change. However, in 2008 the government announced its intention to invest £7 million into walking promotion. This article utilises Kingdon’s Multiple Streams framework as an organising principle through which to interrogate the reasons behind the increased emphasis on walking promotion as part of the public health policy agenda in England.

**Methods:**

The research adopted a case study design. Data were obtained through document analysis of relevant policies and semi-structured interviews with experts in the walking sector, including both government and non-government representatives.

**Results:**

Kingdon’s Multiple Streams theory proposes that at certain points in time, ‘policy windows’ are created through the convergence of a problem, an appropriate solution, and a receptive political environment, and this policy window presents an opportunity for major policy change. The findings of this research suggest that the success of London in securing the 2012 Olympic and Paralympic Games was the primary trigger in the creation of a policy window for walking promotion in recent years.

**Conclusions:**

Despite previous interest in walking promotion from the health and transport sectors, it was the recent alignment with the sports agenda that led to increased political commitment. This raises concerns that the research evidence on the health benefits of physical activity and rising levels of inactivity in England, are insufficient to secure government support and investment, and that multi-sector lobbying and joined-up political action may be critical in advancing this agenda.

## Background

Epidemiological research clearly demonstrates that adults who are physically active have a reduced risk of developing many non-communicable diseases (NCDs) including coronary heart disease (CHD), stroke, hypertension, and type II diabetes [1]. Despite these benefits, modernisation, urbanisation, and advances in technology have led to reductions in physical activity levels globally [2]. In 2011, the World Health Organization (WHO) estimated that more than 30 % of adults worldwide did not engage in sufficient levels of physical activity to benefit their health and prevent disease [3]. Consequently, physical inactivity has been identified as the fourth leading risk factor for premature mortality, accounting for an estimated 6 % of global mortality (3.2 million deaths annually) [4]. In England, recent surveillance data suggests that over 40 % of adults are failing to meet recommended physical activity levels [5]. As a result, physical inactivity is thought to cause 3.1 % of morbidity and mortality in England, and is responsible for 35,000 deaths annually [6].

Brisk walking is a ‘sufficient’ activity to benefit health [7–9] and is viewed as one of the most acceptable and accessible forms of physical activity [10]. Walking is free of charge, does not require specialist equipment or facilities, and can be easily incorporated into everyday life. Walking is an ideal introduction to physical activity for people who are overweight or extremely unfit [11], and being a low impact activity, walking poses relatively few risks of injury [12]. For these reasons, walking has been identified as the form of activity with the greatest potential for increasing the overall activity levels of an inactive population [9, 13] and also as the most likely way that all adults can achieve recommended physical activity levels [14].

There is increasing recognition among physical activity researchers, of the role of policy in addressing population levels of physical inactivity [15, 16]. The development of a national policy framework is important to raise the profile of physical activity as a priority area and to provide a coherent action plan or programme of activities aimed at increasing population prevalence of physical activity [17]. Due to its broad accessibility and acceptability it has been proposed that “walking must be central to any strategy to increase physical activity” [18].

Physical activity and health began to be recognised as an issue requiring government support in England in the early 1990s. The ACTIVE for LIFE campaign, which was funded by the Department of Health (DH), aimed to raise awareness of the health benefits of being active and encourage regular physical activity as part of a healthy lifestyle. The campaign had a strong focus on walking, but was not accompanied by a strategic plan or investment in infrastructure or programs to support physical activity behaviour change.

Despite initial leadership for the physical activity and health agenda from DH, the Department for Transport (DfT) began to recognise the role of walking and cycling in meeting its objectives around reducing congestion and carbon emissions. In 1996 DfT published a *National Cycling Strategy* [19] and announced its intentions to develop a national walking strategy [20]. Although a strategy to promote walking did not emerge until 2004, this also came from DfT in the form of *Walking and Cycling: An Action Plan.*

Physical activity promotion generally, and walking promotion specifically, has the potential to contribute to the aims and objectives of a wide range of government departments. In addition, many of the actions to promote walking fall within the remit of different departments such as health, transport, education, environment, and urban planning. Thus there has been no natural ‘home’ for walking promotion, which has presented challenges to developing a coherent and coordinated national policy.

Since the early 2000s, several non-government organisations have established large scale walking initiatives. For example, the Countryside Agency established the national ‘Walking for Health’ programme (originally known as the Walking the Way to Health Initiative) and also the National Step-O-Meter Programme. These activities were traditionally funded through agencies such as the British Heart Foundation and the Big Lottery, as opposed to the government. However, in 2008 the government announced its intention to invest £7 million in a programme of “innovative campaigns to encourage people to walk more” [21]. This level of commitment and investment in walking promotion was unprecedented and presented a real opportunity for those working in physical activity and walking promotion to develop and deliver large-scale interventions aimed at improving the nation’s health.

In order to move beyond simply a description of this example of policy development, this article turns to the study of policy agendas and considerations of how issues come to be issues in the first place, how agendas change over time, and the factors which determine why some issues are given more government attention than others [22]. The conceptual framework put forward in this article, Kingdon’s Multiple Streams theory [23], serves to shed light on and explain the increased emphasis on walking promotion as part of the public health policy agenda in England. In doing so, this paper aims to answer the following questions:

1. What have been the challenges to developing a coordinated policy on walking promotion?
2. What factors led to the rise of walking on the government’s agenda in 2008?
3. What factors might help maintain the government’s interest and commitment to walking promotion?

## Methods

### Conceptual framework

Due to the complex nature of the policy making process, a range of theories and conceptual frameworks have been developed; these constructs serve the purpose of focusing the policy analyst’s attention on important elements within the policy process, while helping the analyst to apply structure or typologies to an otherwise chaotic and unwieldy course of events. Kingdon’s Multiple Streams framework is particularly focused on the agenda setting process and, as such, lends itself to answering the questions posed in this article [23]. This framework suggests that the policy process consists of three distinct sets of processes or ‘streams’: 1) problems; 2) policies; and 3) politics. At key points in time the three streams are joined—a problem is recognised, an appropriate solution is identified, and the political ‘mood’ is right for the government to embrace and drive forward policy change. This confluence of the three streams is referred to as a ‘policy window’; a juncture at which an opportunity for major policy change can be grasped. Kingdon’s framework is put forward as a useful heuristic device with which to understand agenda setting and policy change.

### Study design

The research adopted a case study design. Therefore the focus was on gaining in-depth insights into the political processes surrounding walking promotion in England, rather than making generalisations about the applicability of the findings to other cases. Data were obtained through document analysis and semi-structured interviews and triangulation techniques were used to verify the validity of the results [24]. The focus of the research was on the period up to October 2012. The study was approved by Loughborough University Research Ethics Committee.

### Document analysis

A literature and web search was undertaken to identify both past and present documents relevant to walking policy in England. The web-search mainly focused on the websites of DH, DfT, and the Department for Culture, Media and Sport (DCMS). Various search terms were used including ‘physical activity’, ‘active travel’ and ‘walking’, and all identified documents were considered. To ensure the comprehensive inclusion of relevant documents, all interviewees were asked to identify documents that they felt were important for understanding the development, content, and/or implementation of walking policy in England. Any documents which had not been previously identified were obtained and included in the analysis. A list of the key documents included in the analysis is provided in Table 1.

**Table 1:** Key walking related documents which were included in the analysis

Document	Author, Year
Saving Lives: Our Healthier Nation	Department of Health, 1999 [36]
Tackling Obesity in England	National Audit Office, 2001 [37]
Game Plan	Department for Culture, Media and Sport Strategy Unit, 2002 [38]
On the Move: By Foot	Department for Transport, 2003 [39]
At Least Five a Week	Department of Health, 2004 [27]
Choosing Health—Making Healthy Choices Easier	Department of Health, 2004 [27]
Walking and Cycling: An Action Plan	Department for Transport, 2004 [40]
The Future of Transport	Department for Transport, 2004 [41]
Choosing Activity—A Physical Activity Action Plan	Department of Health, 2005 [42]
Making the Case: Improving Health through Transport	Health Development Agency, 2005 [43]
Tackling Obesities—Future Choices	Government Office for Science, 2007 [44]
Towards a Sustainable Transport System	Department for Transport, 2007 [45]
Delivering a Sustainable Transport System	Department for Transport, 2008 [46]
Healthy Weight, Healthy Lives: A Cross-Government Strategy for England	Cross Government Obesity Unit, Department of Health & Department for Children Schools and Families, 2008 [47]
Before, During and After: Making the Most of the London 2012 Games	Department for Culture, Media and Sport, 2008
Be Active, Be Healthy	Department of Health, 2009 [48]
Active Travel Strategy	Department for Transport, 2010 [49]
Healthy Lives, Healthy People—Our Strategy for Public Health in England	Department of Health, 2010 [50]
Health Lives, Healthy People—A Call to Action on Obesity in England	Department of Health, 2011 [37]
Start Active, Stay Active: A Report on Physical Activity from the Four Home Countries’ Chief Medical Officers	Department of Health, 2011 [37]
The Public Health Responsibility Deal	Department of Health, 2011 [37]

### Semi-structured interviews

Interviewees were identified using a mix of both purposive and snowball sampling. Firstly, the sampling process involved identifying the key organisations involved in walking promotion in England. The criteria for inclusion were that the organisations had to be recipients of public investment and responsible for managing and/or delivering large-scale walking interventions currently, or within the past five years. Five organisations were identified which met these criteria

(year established in brackets):

1. Natural England (originally the National Parks Commission, 1949);
2. The Ramblers (1935);
3. Sustrans (1977);
4. Living Streets (originally the Pedestrians Association, 1929); and
5. Walk England (2008).

Although each of these organisations is concerned, in some way, with walking promotion, the aims and objectives of each organisation differ and include access to the countryside, pedestrian safety, and transport emissions. The organisations vary substantially in terms of their size and resources; the largest organisations are the Ramblers and Sustrans, while the smallest organisation is Walk England. The primary purpose was not to compare across cases but to consider each organisation’s perspective in order to reach well-rounded conclusions about the development and dynamics of walking promotion as a public health policy issue in England.

Key representatives from each of these organisations were identified using existing knowledge of the organisations and by searching their respective websites. The selected interviewees were either the Chief Executive Officer (CEO) (particularly for smaller organisations) or, if appropriate, the strategic lead for walking and/or health (particularly for organisations with a broader agenda). In addition, DH and DfT have been identified as the ‘main players’ in promoting physical activity for adults [25]. Interviews were conducted with representatives from each of these departments; the interviewees were the Head of Physical Activity and the Head of Active Travel, respectively.

Snowball sampling was used to complement the purposive approach [26]. This involved asking each of the interviewees to identify other colleagues or acquaintances with relevant knowledge and experience who they felt would make a valuable contribution to the research. This approach led to the identification of interviewees from several other organisations including Intelligent Health (a limited company which aims to create physical activity opportunities close to where people live and work), Knowledge into Action (a charity focused on improving health and healthcare), and Sport England (the DCMS funded body responsible for the delivery of sport in England from grassroots to elite level), as well as several independent consultants and other known advocates.

The interview schedule typically included the following themes: how walking fits within the aims and objectives of the different organisations; the roles of the different NGOs within the broad field of walking promotion; which aspects of the broad walking agenda agencies are mostly closely aligned to; how the organisations are funded; who they are accountable to; the main programs that the organisations deliver; relationships/collaborations with other organisations; relationships with government; level of political influence of each NGO; perceptions of how the issue of walking has been dealt with by the government; and barriers to establishing greater political support and investment into walking promotion in England. The interview schedule for the government representatives included questions on: how responsibility for walking promotion has been allocated or dispersed across government; consultation and decision making processes related to the development of walking policy; the main challenges in developing and implementing policy to promote walking; and relationships and interactions with the key NGOs on walking related issues.

Fifteen interviews were conducted in total and took place between April and October 2012. The duration of interviews ranged from 35 min to two hours, and the typical length was one hour. All interviews were recorded on a digital audio device, with consent, and were subsequently transcribed verbatim. Each transcript was sent to the respective interviewee, to confirm that it accurately conveyed what was said or intended. In total, the interview data consisted of 285 pages of transcript.

### Data analysis

Both the documents and the interview transcripts were uploaded into NVivo qualitative software package and analysed using inductive content analysis. Therefore, the coding categories and the names for each concept and theme were derived directly and inductively from the data. The coding themes were then allocated to one of the following three groupings: problems; policies; and politics, in order to analyse the results in relation to Kingdon's Multiple Streams framework [23]. To confirm the reliability of the analysis, all data were coded on two separate occasions, allowing the lead researcher to confirm or refine the coding system developed during the initial analysis.

## Results

The following section is set out according to the broad, yet distinct categories in Kingdon’s Multiple Streams framework: problems; policies; and politics. The article focuses on events and decisions taking part in each of these ‘streams’ before considering how these factors have converged to make a ‘policy window’ for increased support and investment in walking promotion in England.

### The problem stream

Walking levels in England have been in decline since the mid-1970s and this reduction in walking has been accompanied by an increase in car use [27]. The consequences of this shift include reduced overall physical activity levels, increased traffic congestion, and higher levels of carbon emissions. The problems associated with low levels of walking have been recognised by several well established interest groups/organisations, which formed a key focus of the empirical research. These types of interest groups play an important role in nearly every aspect of health policy, from bringing issues to the attention of government, proposing new policy options, and building pressure for action [28].

It is imperative that issues are defined in a way which will attract political interest. According to Weiss [29], issue definition is concerned with the organisation of a set of facts, beliefs, and perceptions, or ‘how people think about circumstances’. The way in which an issue is ‘packaged’ determines how it is perceived by both policymakers and the public and thus can impact upon the agenda-building process [30]. ‘Symbols’, which can be described as “objects to which people attach political significance”, are used to attract attention to an issue, to define an issue in a specific way, and to mobilise support for specific policy options over others [30]. The issue of walking promotion has been defined or ‘packaged’ in three primary ways: as a health issue; a transport issue; and as an environment issue; and this has impacted on how responsibility for walking promotion has been dealt with by the government. A senior staff member from the Ramblers stated, for example:

“I think it has been spread between transport and health and environment… and it’s kind of shifted and moved around depending on whether you’re talking about the countryside or whether you’re talking about urban walking, or obesity or issues like that” (London, May 2012).

Sometimes recognition that a problem exists is sufficient for the problem to make it onto the political agenda; however there are usually many problems competing for recognition, meaning that only a fraction of them make it into the formal process of political deliberations. Which problems receive government attention is often influenced by ‘policy entrepreneurs’ [23, 31]. These entrepreneurs are highly motivated individuals who seek to raise the profile of an issue among both government officials and the general public. Policy entrepreneurs typically hold positions of leadership within relevant interest groups and are usually well connected politically. The main roles of an entrepreneur are to define and reframe problems, advocate new ideas, specify policy alternatives, broker ideas among policy actors, mobilise public opinion, and help set the decision-making agenda [32].

There have been several long standing advocates for walking promotion, who have been instrumental in bringing the issue to the attention of government and for encouraging political action. These include Dr William Bird, a general practitioner who was instrumental in the establishment of the national led walk program Walking for Health, and Sir Muir Gray, who has held several senior positions in preventive health and has been described as a “a ceaseless champion of walking as a means of tackling obesity and inactivity” [33]. A former employee at DH reflected on the powerful influence of these types of policy entrepreneurs:

“They can walk the talk. They brought good examples of what was happening elsewhere… you talk about people being influential and stuff like that. It's a fact of life that certain people will like other people and listen to what they say. And it happens more than you could ever believe in terms of someone having the ear of a Minister” (London, July 2012).

The lobbying efforts of these policy entrepreneurs have been facilitated by several factors including growing research evidence on the health benefits of walking [9, 10] and prevalence data on rising levels of inactivity, for example from the ‘Allied Dunbar Fitness Survey’ [34], and more recently the Health Survey for England [5, 35]. One of the biggest challenges for these policy entrepreneurs, however, has been to convince policymakers that walking promotion legitimately falls within the government’s remit. There is a long history of policy in England which emphasises the importance of individuals taking responsibility for their own health behaviours. For example, *Saving Lives: Our Healthier Nation* [36], identified behavioural risk factors such as smoking and physical activity as an individual responsibility and beyond the remit of the government. Even some of the more recent policy documents, including *Healthy Lives, Healthy People—A Call to Action on Obesity in England* [37], emphasise the need for individuals to take responsibility for their own health by making healthier lifestyle choices. Therefore the challenge has not only been to convince the government of the magnitude and consequences of the problem of low walking levels but also to convince them that dealing with the problem is a government responsibility.

An additional barrier to walking promotion, which was expressed by representatives from both DH and DfT, is the perception that walking is such a simple behaviour that the general public will not view walking promotion as sufficiently complex or necessitating high level expertise, to warrant political attention, and thus this will not be considered an appropriate use of scarce government resource. This sentiment is captured by the following quote from a senior government official:

“Governments can feel a little foolish promoting walking in a sense that it's a Daily Mail headline—Government tells people to walk!—Government gives people lessons on walking! Suddenly you can be ridiculed because it’s such a natural thing to do” (London, April 2012).

### The policy stream

The linking of solutions to policy problems is thought to increase the chances of gaining political attention and support for an issue. Having pre-formulated policy solutions can increase the government’s confidence that there are appropriate solutions to the identified problem and thus that the problem can be dealt with in a timely fashion without the need for drawn-out political deliberations on appropriate policies. Therefore once one or more problems are identified, ‘policy communities’, consisting of experts in the area, try to affix solutions to the problem, usually driven by their own values and interests [38].

Each of the key walking organisations has conceptualised different policy solutions, including led walk schemes, infrastructure changes to improve the environment for walking, and resources such as websites and maps. Multiple Streams theory holds that the survival of ideas and solutions in the policy stream is determined by three factors.

- First, the degree of technical feasibility, which relates to how easily a theoretically sound idea can actually be translated into practice. Ideas that can make the transition from theory to practice with the least difficulty are thought to stand a better chance of survival.
- Second, survival is determined by whether solutions are widely supported by a range of specialists within the policy community. The more wide-spread support there is for a policy solution, the greater the likelihood that the solution will be adopted.
- The third factor relates to budgetary implications, with less costly solutions often receiving a greater level of support from policymakers [22].

In recent years two walking programmes have received substantial government resource; Natural England’s Walking for Health programme and Walk England’s Walk4Life Miles project, which received £3 million and £1.4 million respectively from DH in 2008. Natural England’s Walking for Health programme is a led walk initiative, established in 2000. Walking for Health had already expanded into a national programme and in 2010 the programme consisted of over 600 local schemes, all of which were delivered by a network of over 11,000 trained volunteers [39]. This programme, in many ways, met Kingdon’s proposed criteria for survival within the policy stream. Walking for Health had a proven track record of feasibility, the programme had widespread support from various stakeholders (and particularly Dr William Bird), and it could be delivered at relatively low cost due to the engagement of a large network of (existing) volunteers.

Walk England’s Walk4Life Miles project was a new initiative which would involve setting up 2012 one mile sign-posted walking routes across the country. It was envisaged that the one-mile routes would be safe, attractive, and connected to where people live, and that people would be able to use the miles to test their fitness, using the principles of the Rockport One-Mile Walk Test [40]. The aim of the project was to get 30,000 people to improve their fitness and sustain an increase in physical activity [41]. The simplicity of this intervention would facilitate judgements of feasibility and cost and, although there was not wide spread support for the initiative from the walking sector as a whole, it was lobbied for fiercely by Walk England, as illustrated by the following quote:

“I found out that Walk England had snaffled a million quid… the reason that happened was that [they] never got off the phone from [the Department of Health]. They badgered, badgered, badgered, badgered and badgered. And just badgered [the Department of Health] so badly that in the end that’s what happened” (London, May 2012).

Although the ‘evidence-based policy movement’ has sought to promote the rigorous analysis of policy options in order to improve decision-making [42, 43], the findings of this research lend support to Head’s suggestion that policy development is often based more on politics and professional judgement, rather than on research evidence alone [43], and highlights the influence that key ‘policy entrepreneurs’ can have in the decision making process.

### The politics stream

The politics stream relates to the political ‘mood’ and openness to change based on the current political climate [22, 23]. Clearly a range of factors such as impending elections, a change in government, and interest group activity can lead to the inclusion or exclusion of different topics on the political agenda, as well as influencing how these problems are perceived by the electorate and policymakers, and how potential solutions are evaluated.

In July 2005 it was announced that London would host the 2012 Olympic and Paralympic Games. Subsequently DCMS released *Before, During and After: Making the Most of the London 2012 Games* [21], which outlined the Government’s intention to make the UK a world-leading sporting nation. However a key feature of both the bid and the subsequent policy was the promise of delivering a ‘physical activity legacy’ which would inspire population increases in sport and physical activity, or, as one interviewee summarised it, political interest in physical activity and walking promotion was bolstered by the world’s largest sports mega-event: “The driver, I would say, was the Olympics, because funding was allocated to help meet that target” (London, July 2012).

Specifically this policy identified the target of getting two million more people ‘active’ by 2012, and committed to investing £7 million into walking promotion as a key approach to achieving this target. Interestingly, the basis of this legacy is the belief that elite sport success can act as a catalyst for increased physical activity and sport participation among the masses; a belief that has little evidence from previous sports mega-events [44–47].

### The ‘policy window’

At key points in time the three streams outlined above are joined together: a problem is recognised, an appropriate solution is identified, and the political ‘mood’ is right for the government to embrace and drive forward policy change. This confluence of the three streams is referred to by Kingdon as a ‘policy window’.

The success of London in securing the 2012 Olympic and Paralympic Games was the primary trigger in the creation of a policy window for walking promotion in recent years. The profile of hosting this mega-event meant that political interest was high, and the subsequent promise of delivering a physical activity legacy provided a ‘problem’ in that the government were now required to provoke large scale increases in physical activity [21, 48]. Time and resources were allocated to delivering this target and thus the government were seeking appropriate policy solutions in which to invest. A former employee at DH recollected on this situation:

“We had a target to meet and we had to get two million people active so we had to find programmes that would do that and it was very clear that the biggest potential was in walking. I think what possibly wasn’t clear was what the right interventions were” (London, July 2012).

Therefore, the role of interest groups and policy entrepreneurs was to identify appropriate policy solutions and to convince the government of their value. Two organisations were successful in this endeavour, Natural England and Walk England.

A key feature of the policy window however, is that as quickly as it opens, it may close, due to other competing agendas or simply a change in the political ‘climate’. In May 2010 there was a general election and a change in government. When the new government came into power, the UK (and the rest of the world) was in the midst of an economic recession. In an attempt to address the economic crisis the coalition government undertook a review of non-departmental public bodies, including Natural England. The review concluded that Walking for Health was peripheral to Natural England’s core objectives and was not something that it should be delivering. A competitive tendering process ensued and in March 2012, the Ramblers took over the coordination of the Walking for Health programme [49].

In addition, there was a Treasury review of the public spending commitments made by the previous government between 1^st^ January 2010 and the General Election [50]. This review examined £34 billion of spending that was approved during the previous government’s final few months in office. The aim of the review was to assess whether these commitments were affordable, whether they would deliver value for money, and whether they were considered a priority for the new government. In total 12 projects were cancelled because they were deemed unaffordable and not a government priority, one of which was Walk England’s Walk4Life Miles project. This is an example of the ‘window of opportunity’ closing due to a change in politics.

## Discussion

This article reports on the application of Kingdon’s Multiple Streams theory to explain the recent rise of walking promotion on the political agenda in England. The framework provided a useful structure for the study of agenda setting in this discrete area of policy, thus reinforcing the utility and wide applicability of Kingdon’s Multiple Streams theory for policy analysis.

The analysis identified the London 2012 Olympic and Paralympic Games as the primary driver behind the government’s increased interest in walking promotion in England. Both the 2012 Games bid and the subsequent policy used the rhetoric of inspiring population level increases in physical activity. It is interesting to note that despite a long-standing interest in walking from DH and DfT, it was alignment with the sports agenda that led to increased investment in walking promotion in recent years. This is supported by the concept of ‘generalisation of interests’ which proposes that if policy entrepreneurs are able to demonstrate the relevance of an issue to a broad audience (and a range of government departments), this increases the appeal of the issue and the likelihood of securing government engagement and support [51, 52].

The £7 million investment into walking promotion in 2008 was motivated by the perceived potential of sports mega-events to lead to population level increases in sport and physical activity participation; however, there is little evidence to support this notion [44–47]. Hosting sports mega-events does, however, generate political interest and thus sport can be a strong ‘symbol’ for getting physical activity onto the political agenda. Therefore further research is needed to understand how these events may be better utilised as a vehicle for encouraging mass participation in sport and physical activity. It should be noted that linking physical activity promotion with sports mega-events alone is insufficient, as it fails to recognise the importance of physical activity as a critical lifestyle behaviour for the prevention and control of NCDs. In addition, this sort of interest and commitment is often short-lived and is not sustained beyond the event itself. In the case of the London 2012 Games, the target of getting two million more people active as a result of hosting the Games was dropped even before the event took place [53, 54].

Following the government’s promise to deliver a physical activity ‘legacy’ as a result of hosting the Games, it committed to investing in a suite of “innovative campaigns to encourage people to walk more” [21]. The government elected to fund two walking initiates; Natural England’s Walking for Health programme and Walk England’s Walk4Life Miles project, neither of which were supported by a robust scientific evidence base.

Although there is some evidence that walking in groups is an effective approach to increasing physical activity levels [55], relatively little is known about the effectiveness of other approaches to encourage people to walk more. The National Institute for Health and Care Excellence advocate for action to promote both leisure and transport related walking [14], through a range of portfolios including leisure services, parks, transport, and the environment, however further research is needed on exactly what types of interventions are effective and cost-effective.

In the absence of strong evidence of effectiveness, the lobbying efforts of policy entrepreneurs will be particularly critical. In addition it is advantageous to package interventions in a way which attracts political interest and aligns with other government priorities. In recent years, Walk England were particularly successful in this regard; the concept of 2012 routes gave this intervention a (albeit loose) connection to the 2012 Games and provided DH with a clear policy solution linked to the legacy target. Thus although the initiative was innovative, lacked a sound theoretical or empirical evidence base, and did not meet Kingdon’s criteria of having wide-spread support, Walk England were able to secure government investment.

Further research is clearly needed to build the evidence base on effective walking interventions. In the meantime, lobbying for interventions which lack evidence of effectiveness should be undertaken with caution. If these programs do not lead to the desired outcome in terms of increasing physical activity levels, they will have the adverse effect of undermining the government’s trust and confidence, which is likely to lead to reductions in future support and investment.

Evidently there is still work to be done to a) raise awareness of the health benefits of physical activity; b) emphasise the importance and potential of walking promotion for influencing population levels of physical activity; c) build the evidence base on effective approaches to promoting walking; and d) encourage the development and implementation of policy level actions, with sustained support and investment, to increase population levels of physical activity and reduce NCD prevalence.

## Conclusion

This paper utilised Kingdon’s Multiple Streams framework as an organising principle through which to interrogate the reasons behind the government’s increased interest and investment in walking promotion in 2008. Overall it appears that government interest in walking promotion in England has largely been motivated by sport and the promise of delivering a ‘legacy’ as a result of hosting the London 2012 Olympic and Paralympic Games. This raises concerns that the research evidence on the health benefits of physical activity and rising levels of inactivity in England, are insufficient to secure government support and investment, and that multi-sector lobbying and joined-up political action may be critical in advancing this agenda.
